# Health and Economic Impact of COVID-19: Mapping the Consequences of a Pandemic in Malaysia

**DOI:** 10.21315/mjms2020.27.2.16

**Published:** 2020-04-30

**Authors:** Sadia Shakeel, Mohammad Azmi Ahmed Hassali, Atta Abbas Naqvi

**Affiliations:** 1 Discipline of Social and Administrative Pharmacy, School of Pharmaceutical Sciences, Universiti Sains Malaysia, Pulau Pinang, Malaysia; 2 Faculty of Pharmaceutical Sciences, Dow University of Health Sciences, Karachi-Sind, Pakistan; 3 College of Clinical Pharmacy, Imam Abdulrahman Bin Faisal University, Dammam, Saudi Arabia

**Keywords:** Malaysia, coronavirus, outbreak

## Abstract

The World Health Organization (WHO) has termed the novel coronavirus infection a pandemic based on number of confirmed cases in more than 195 countries and with risk of further spread. The infection has had drastic impact on global trade and stock markets. The Malaysian authorities realised the need to ensure availability of health resources and facilities in the country so that the healthcare professionals could treat serious cases on priority basis. Steps have been taken to ensure that health facilities are not overwhelmed with cases and do not become the source of virus spread to other healthcare staff and patients.

## Virology, Transmission and Symptoms of Infection

**Figure 1:**
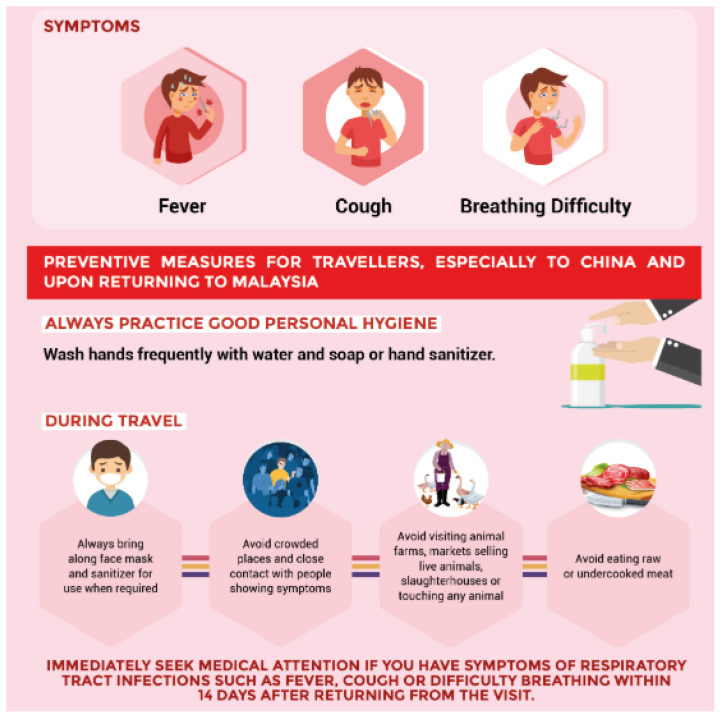
Symptoms of COVID-19 and preventive measures Source: Ministry of Health Malaysia

Coronavirus (CoV) is a family of viruses and could be divided into four major sub-groups, recognised as alpha, beta, gamma and delta. Human beings usually get sick when infected with human CoVs; 229E, NL63, OC43 and HKU1. These viruses are zoonotic as they may be transmitted between animals and humans (1). At times, CoVs that infect animals mutate into a new human CoV. The recent examples are Severe Acute Respiratory Syndrome (SARS-CoV) and Middle East Respiratory Syndrome (MERS-CoV) that had the symptoms of common cold, fever, malaise and in some cases, severe shortness of breath and pneumonia. Evidence indicates that SARS-CoV was transmitted from civet cats and MERS-CoV from dromedary camels, to human beings (2). Few other identified CoVs are present in animals but have not infected humans yet. Previously the novel coronavirus (nCoV) was an unknown strain that was not reported in humans (3). The coronavirus disease (COVID-19) is not similar to any other CoVs that usually infect humans and become a source of mild sickness or common cold. A diagnostic finding with CoV 229E, NL63, OC43 or HKU1 is not similar to a COVID-19 diagnosis and patients with COVID-19 are treated differently as compared to those infected with common CoV diagnoses. The risk associated with outbreak is dependent on features of the virus such as its human transmission, contagiousness as well as the intensity of symptoms. It is further dependent on the therapeutic actions taken to prevent/reduce the intensity of disease, i.e. vaccine or medicines (4). Fever, shortness of breath, cough, pulmonary symptoms and difficulties in breathing are commonly observed symptoms. However, an infection may become the reason for SARS, pneumonia, renal failure and even death in more severe cases. The standard recommendations for prevention of infection spread include washing hands regularly, covering mouth and nose while sneezing or coughing, cooking eggs and meat properly, and limiting interaction with anyone showing signs of respiratory disease (5) (Figure 1).

## A Large Spike in Cases in March 2020

The World Health Organization (WHO) declared COVID-19 a pandemic on 11 March 2020 and feared that it could affect every individual and further mentioned the need for a joint effort (6). The WHO advised Malaysian health authorities to be prepared for a larger spread of infection. It further advised that since the virus may be more contagious than assumed, it requires protecting vulnerable population as a priority. At the same time, action is required to minimise its impact on health and social well-being. For instance, in case of increased community transmission, the health authorities would need to ensure that health facilities can treat most susceptible and serious cases (7). The health strategy of Malaysian authorities transcends from isolating or quarantining everyone who is infected, to encouraging people with the mild illness to stay at home. This strategy is beneficial as health care facilities would not be overwhelmed and may not become the source of virus spread to other healthcare staff and patients.

Instead of testing every suspected case and tracing contacts of those who are infected, Putrajaya health administration planned restricted testing to screen geographical spread and patterns as well as utilise information to settle on informed choices on public health response. It is important for Malaysians to care about their well-being and not to follow unauthentic literature as it may be harmful (7).

Since the COVID-19 outbreak is larger than SARS or MERS-CoV outbreaks, it requires intensive actions (8). China has successfully limited the spread of virus while other countries namely Japan, Singapore and Hong Kong have also had some success in limiting the pandemic. It is pertinent to mention that previous experience of dealing 2003 SARS epidemic may have contributed to their success. Conversely, most countries in Europe and the US are not being able to limit the spread as they had no previous experience with dealing with an outbreak. As a consequence, the epidemic is now intensifying across these countries (9). Pharmaceutical companies have been in the focus lately as they are developing new vaccines and medicines for prevention and treatment of COVID-19, respectively (10, 11).

Malaysia has the largest number of confirmed cases in Southeast Asia. The country reported its first death owing to COVID-19 on 17 March 2020. The first human-to-human transmission of COVID-19 was identified on 12 March 2020 raising concerns of local spread (11). Most new infections in the country were linked to religious congregation. Malaysia is currently limiting the nationwide movement to restrict the spread of COVID-19. It is further prohibiting all visitors and residents from traveling abroad, closing all places of worship, schools and business premises excluding essential items businesses and marketplaces that supply items of daily needs. The measures are effective from 18 March 2020 onwards. At the same time, Malaysians returning from abroad must go through a 14-day self-quarantine (12).

## Influence on Economy, Trade and Tourism

**Figure 2:**
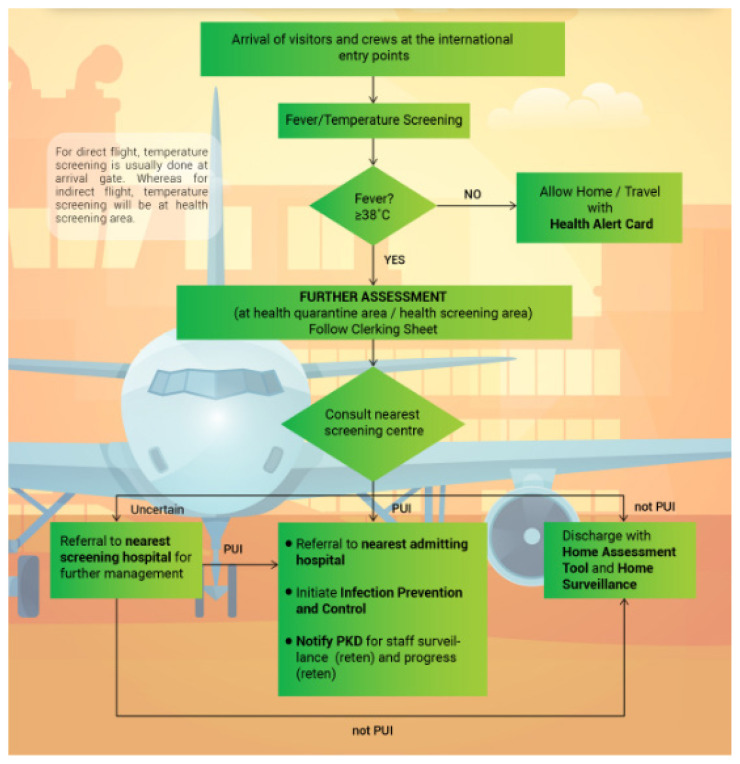
Flow chart for screening of travelers in Malaysia Source: Ministry of Health Malaysia

The Visit Malaysia 2020 (VM2020) campaign that aimed to attract 30 million tourist arrivals has also been cancelled due to COVID-19 crisis. It is of concern as the tourism industry of Malaysia is a major source of foreign exchange in national economy. The tourism contributes to more than 50% of export trade-in service. Approximately 27 million tourists visit in Malaysia annually. Tourists are recommended to fulfill additional screenings requirements implemented by Malaysian authorities as East Malaysia is a tourism hotspot for Chinese citizens (Figure 2). For instance in state of Sabah, the Chinese travelers constituted 44% of all visitors in 2018. Over 2010–2018 period, tourism in Malaysia from China including Hong Kong and Macao rose by 160%, outpacing the 5% development pace of visitor arrivals. On a compound average growth rate (CAGR), visitors from China increased by 12.7% per annum while visitors from other places increased by 0.6%. The visitors from China increased from 4.6% in 2010 to 11.4% in 2018 (9). This increase in number of visitors from China was also due to ease in travel advisories such as availability of electronic travel registration and information (eNTRI), visa-free accesses, visa on arrival (VOA) and e-visas. The government estimated the VM2020 campaign to attract 30 million tourists and revenues up to RM 100 billion. However, with COVID-19 crisis and 10.6% of the estimated target being visitors from China, it would seriously decrease the number of visitors and revenue. During 2003 SARS outbreak, visitors from China decreased by 37% whereas total number of tourists decreased by 21%. As a consequence, the revenue from tourism decreased by 39% for visitors from China and 17% for visitors of other nationalities. Considering the magnitude of current outbreak, the numbers could be more detrimental for the Malaysian economy in 2020 (12–13).

According to the International Air Transport Association, monthly international traveler circulation resumed to its pre-SARS outbreak level within nine months. Since COVID-19 outbreak is much greater in scale, it is possible that resumption of pre-COVID-19 travel circulation level would take more than nine months (13). Though, the services sector including tourism industry, which accounts for nearly 57% of Malaysian economy has remained stable. In the first nine months of 2019, it expanded by approximately 6.1% and contributed roughly 76% of Malaysian GDP growth. However, the ongoing outbreak of the COVID-19 has proved quite challenging (14).

**Figure 3:**
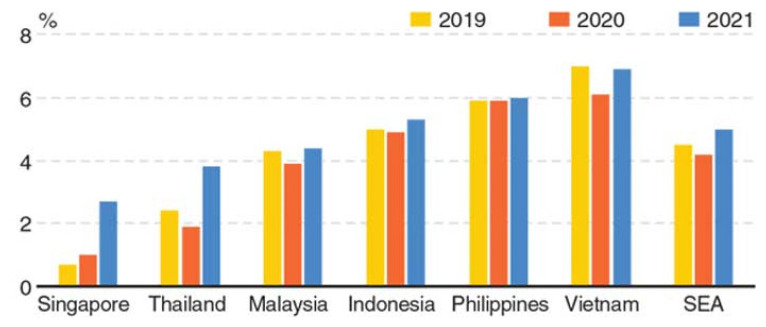
GDP forecasts of some Asian countries Source: Oxford Economics/Haver Analytics

At the same time, retail market and accommodation significantly add to revenues along with tourism. They contribute about 15% of the services sector together. Even though visitors’ expenses are not directly considered as domestic consumption, these expenses significantly add to the economy as expanded services may promote consumer-linked businesses together with exports. This would eventually stimulate domestic spending. Considering the likelihood that tourism will decrease in the year 2020, Malaysia’s private consumption growth will be affected. This may prove to be burdensome for the economy as private consumption alone was more than half of the economy and contributed over 90% of significant development in nine months of 2019 (9M2019) (12). Malaysia’s financial development has scaled down to 4.3% in 2019, the lowest since 2016 and below the previous lowest growth rate of 5.4% recorded in 2010. Financial development in Q4 2019 was 3.6%, the lowest in 10 years. The Bank Negara Malaysia, which is the central bank, declared that the COVID-19 would negatively impact Malaysia’s financial development. In this regard, several measures such as reduction in statutory reserve ratio (SRR) by 100 basis points to 2% and release of RM30 billion, i.e. USD6.81 billion into the financial framework, were taken (14) (Figure 3).

We call on other researchers to provide their views on the socioeconomic consequences of COVID-19 and present recommendations to navigate through this crisis.
